# Experience of residents learning about social determinants of health and an assessment tool: Mixed‐methods research

**DOI:** 10.1002/jgf2.559

**Published:** 2022-05-15

**Authors:** Junki Mizumoto, Daisuke Son, Masashi Izumiya, Shoko Horita, Masato Eto

**Affiliations:** ^1^ Department of Medical Education Studies, International Research Center for Medical Education, Graduate School of Medicine The University of Tokyo Tokyo Japan; ^2^ Department of Community‐based Family Medicine, School of Medicine Tottori University Faculty of Medicine Yonago Japan

**Keywords:** assessment tool, mixed‐method study, postgraduate learning, reflection, social determinants of health

## Abstract

**Background:**

Educating healthcare professionals about the social determinants of health is important in improving health outcomes of marginalized patients. Residents' experience of learning about the social determinants of health and a clinical assessment tool remains unclear.

**Methods:**

Residents participated in an online session about the social determinants of health and the assessment tool. Using the New World Kirkpatrick Model, we obtained data about participants' experience from various perspectives. The data were analyzed using a concurrent triangulation mixed‐methods design.

**Results:**

The study included 20 out of 30 residents. Their response was good, and self‐reported learning scores were increased by the session. They learned when to ask about patients' social conditions, what to ask, and how to coordinate medical care appropriately. Participants reported reflecting on their role as medical professionals and implementing new practices based on their learning, as well as concerns about addressing patients' social conditions.

**Conclusion:**

Through learning about the social determinants of health, and assessment tools, residents both acquired knowledge and skills, and reflected on their previous practice, accepted patients as they are, understood difficult patients better, and developed interprofessional collaboration. Medical education about the social determinants of health can trigger changes in residents' views.

## Graphical abstract

Through learning about the social determinants of health and an assessment tool, residents not only acquired knowledge and skills, but reflected on their previous practice, accepted patients as they are, understood difficult patients better, and developed interprofessional collaboration. Medical education about the social determinants of health can trigger changes in residents’ views.

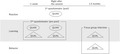

## 1 INTRODUCTION

Social determinants of health (SDoH) play an important role in primary healthcare services. Social factors surrounding patients will influence the effectiveness of clinical treatments, and healthcare professionals may need to address social and structural issues that will negatively affect patients' health.^1^ Healthcare professionals may, however, find it difficult to ask patients about their social conditions^2^ because it is different from asking about medical problems.^3^ To contextualize patient care to support patient health and deliver equitable medicine, healthcare professionals must know how to address patients' social issues appropriately.^4^

Education about SDoH for healthcare professionals should be designed to improve the quality of healthcare for underserved individuals, communities, and populations.^5^ The purpose of education about SDoH includes being aware of patients' social problems and needs, and organizing appropriate care in clinical settings. Learning about SDoH and a simple screening tool with some practices helped residents acquire knowledge and encouraged them to document patients' social problems.^6^, ^7^, ^8^, ^9^ It is still unclear, however, how trainees experience learning about SDoH assessment tools and practicing tool‐guided care. Most previous research has also been conducted in the outpatient setting, and the effects of education in other settings should also be examined.

In Japan, 2 years of postgraduate training is practically mandatory for medical graduates. Transitional residency programs are offered by various hospitals in Japan, including small‐scale community hospitals. Residents (doctors in the first 2 years after graduation) in small‐scale community hospitals are potential candidates for primary care physicians. They often see patients with various difficulties and need to use interprofessional collaboration and understand patients' social contexts.^10^ SDoH is too complex to teach through traditional didactic methods, and community‐based experiences are more suitable for developing new insights and deeper understanding.^11^ Residents in small‐scale community hospitals would therefore be desirable research participants to clarify the outcomes and mechanisms of specific education on SDoH.

This study aimed to answer three research questions:

RQ 1. What is the experience of residents learning about SDoH and an assessment tool, and what kind of changes does it cause?
RQ2. What will the residents who learned about SDoH and an assessment tool experience in their daily clinical practice afterward?
RQ3. How do residents perceive an assessment tool for SDoH?

## 2 METHODS

### 2.1 Study design and setting

The participants included residents, whose postgraduate year (PGY) was 1 or 2, in nine teaching hospitals. The considered hospitals were small and provide community healthcare services. The federation proclaims a philosophy of nondiscriminatory and equitable medical care. The first author provided a session about SDoH by request from the branch. The residents were all obliged to participate in the session, but they were told that their participation in this study was voluntary, and nonparticipation would not cause any disadvantage. All of the residents were recruited to the research. There were no exclusion criteria. This research was approved by the Research Ethics Committee of the University of Tokyo Graduate School of Medicine and Faculty of Medicine (No. 2020389NI). We obtained written consent from all participants before the study. The session was held online in December 2020 because of the COVID‐19 pandemic.

### 2.2 Designing and implementing the session

The objective of the session was to familiarize residents with the concept of SDoH and help them to address the social issues of their patients, to support their delivery of contextualized medical care. To achieve this objective, we needed to provide a specific clinical tool, ‘social vital signs’ (SVS), that would enable the residents to assess patients' social conditions and develop interventions. This sheds light on the importance of always asking about and assessing patients' social conditions.^12^, ^13^ To assess complex information about SVS and share it with other professionals, professionals often use a matrix sheet to record each social factor to ask (see File 1 in Appendix S1).

We designed the session based on Mezirow's Transformative Learning Theory.^14^ This theory describes how a striking and shocking experience, or disorienting dilemma, can challenge a learner's unconscious beliefs. This causes learners to critically reflect on their frame of reference and acquire new perspectives.^15^ Transformative learning is thought to have a high affinity with education in SDoH and can be used to design training and measure learning outcomes.^5^ To trigger a learning transformation, we constructed the session in five steps. (1) We provided an illustrative case of a patient with seemingly selfish behavior, and asked participants to analyze their emotions towards this patient and consider their next action. (2) We explained SDoH and the assessment tool, and presented the social background of the patient. (3) We asked participants again how they would respond to the patient. (4) We had participants to recall “difficult patient encounters” that they had experienced and filled in the sheet based on those patients. (5) Participants shared thoughts and impressions. We finalized the design of the session with some modifications in response to advice from the team that developed the concept of SVS.

### 2.3 Evaluating outcomes

**FIGURE 1:**
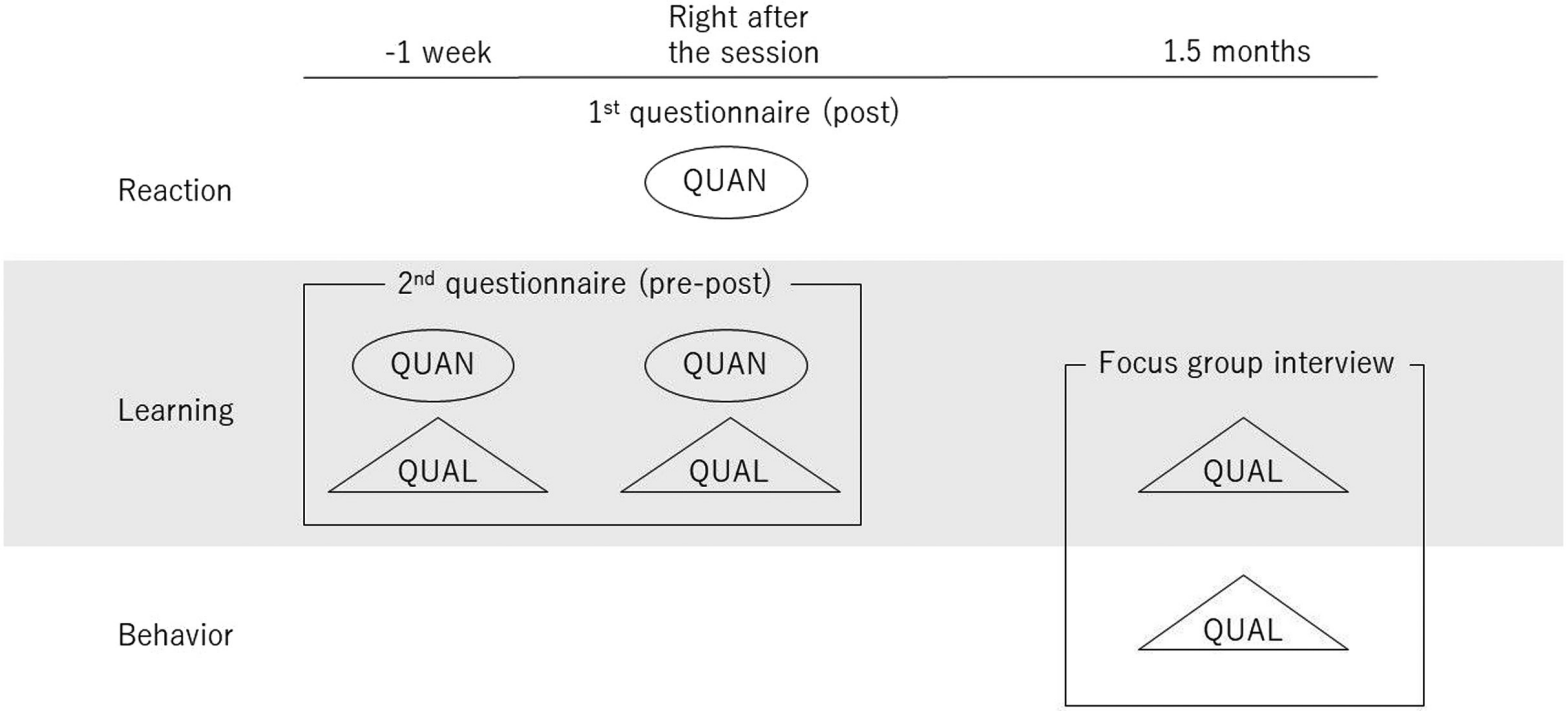
Overall study design and outcome evaluation. Abbreviations: QUAL, qualitative data; QUAN, quantitative data

We evaluated educational effectiveness based on the New World Kirkpatrick Model, which has four levels of training evaluation: reaction, learning, behavior, and results.^16^ We wanted to obtain data from various perspectives at multiple levels, gain thorough understanding, and then prioritize answering the research questions. We therefore used a mixed‐methods approach based on the paradigm of pragmatism.^17^ To our knowledge, a validated questionnaire for assessing physicians' competency about patient care related to SDoH has not been published. We inferred that only quantitative analysis did not cover all of the potential educational contents. We thus collect both quantitative and qualitative data complementarily and followed a concurrent triangulation mixed‐methods design in which we compared, integrated, and interpreted the numeric data at the reaction and learning level and the qualitative data at the learning and behavior level.^18^ Figure 1 shows the overall design of this evaluation. We assessed participants' immediate reactions to the session using a six‐question questionnaire with a five‐point Likert‐type scale (1: strongly disagree to 5: strongly agree) and a blank space for free comments. This questionnaire was specific to the session and developed drawing on prior literature.^19^, ^20^ We distributed this questionnaire by email soon after the session.

Second, we assessed participants' learning just after the session using a questionnaire with six questions based on a five‐point Likert‐type scale (1: strongly disagree to 5: strongly agree), three open‐ended questions, and a blank space. This questionnaire was also specific to the session and developed through a focus group interview with the team who developed the SVS. We selected nine team members as interviewees from a variety of backgrounds: six doctors, one nurse, one medical social worker, and one medical clerk. The interview was started by sharing some example items from previous studies.^6^, ^19^, ^20^ The interviewees were then asked to discuss what participants were expected to learn from the session. They insisted that both understanding of SDoH and acquisition of clinical skills should be underlined. All the interviewees approved the final version. These steps were to improve the content validity of the questionnaire. We distributed this questionnaire twice, before and soon after the session, by email. These questionnaires were answered anonymously. All questions were developed and answered in Japanese, and then translated into English for this paper.

Third, we assessed participants' learning through changes in their daily practice and behavior, using focus group interviews approximately 1 and a half months after the session. The interviews consisted of semi‐structured inquiry about the effectiveness of the session and its impact on daily clinical practice. The first author was in charge of the interviews. The interview guide is shown in File 2 in Appendix S1.

### 2.4 Data analysis

We used mean and standard deviation (SD) to summarize the answers from the Likert‐type scales. To measure the outcomes of learning just after the session, we used a paired‐sample t‐test between the pretest and post‐test scores. Cohen's d was calculated to preliminarily measure effect size,^21^ and Cronbach's alpha to preliminarily evaluate reliability of the questionnaire about the responses.^22^

We analyzed the contents of the open‐ended answers about participants' learning using data‐driven thematic analysis.^23^ A joint display was constructed to integrate quantitative and qualitative data. We also recorded the interviews about participants' learning and behavior, and the audio data were transcribed verbatim. We analyzed these data using the Steps for Coding and Theorization (SCAT).^24^, ^25^ This has four steps: finding noteworthy words or phrases; paraphrasing; drawing out concepts; and developing themes and constructs to fit the context.^26^ The first author conducted these coding processes, and the second author reviewed the coding. They then discussed and modified the coding. The third, fourth, and fifth authors carried out additional checks and modification. All participants then checked the concepts and themes that had emerged. These qualitative analyses were based on the theory of social constructivism. We performed every interview in Japanese. We coded in both Japanese and English iteratively and completed the theme lists in both languages. Researcher reflexivity was shown in Table 1.

**TABLE 1:** Researcher reflectivity

The first author, who is a family physician and Ph.D. student in medical education, was the instructor of the session and the interviewer, and had already known about the participants through regular educational events. To avoid an undesirable authority gradient between the participants and the instructor, participants were explained repeatedly that research participation was voluntary and they could withdraw from the research at any time. The third author collected and anonymized the questionnaire, and participants were explained that the first author could not identify the respondent of each questionnaire. During each interview, the interviewer tried to encourage participants to verbalize their learning and experiences by referencing shared context and good learner‐educator relationships.
Other authors did not engage in the educational session directly. Their engagement was to develop research design, collect and analyze data, and revised the manuscript, together with the first author. The second author is a family physician and expert in medical education research and completed the qualitative analysis with the first author. The third, fourth, and fifth authors are experts in medical education research and revised the results of the analysis. Through the research, these four co‐authors were aware that they should check the relationship between the first author and the participants and, if necessary, suppress the first author's excessive involvement in the relationship.

## 3 RESULTS

Overall, 20 out of 30 residents (66.7%) agreed to participate in the study, of which four were female and 12 were in their first postgraduate year.

### 3.1 Response

In the first questionnaire (response; post‐survey), the mean scores of the items ranged from 4.35 to 4.65 (see Table 2). Cronbach's alpha was 0.77.

**TABLE 2:** Mean scores for responses after the session (first questionnaire)

Item	Mean score (95% CI)
The information about SDoH and SVS made me reflect deeply on my daily clinical practice.	4.6 (4.36–4.84)
There was adequate orientation in taking patients' SVS.	4.35 (4.00–4.70)
I think this session was suitable for our residency programs.	4.35 (4.00–4.70)
I think I want to introduce social vital signs into my daily practice.	4.6 (4.32–4.88)
I think I want to know more about SDoH and SVS.	4.35 (4.00–4.70)
I feel satisfied with this session.	4.65 (4.38–4.92)

Abbreviation: CI, Confidence interval.

### 3.2 Learning immediately after the session

In the second questionnaire (learning immediately after the session; pre–post survey), five out of six items showed a statistically significant increase in the mean score post‐test compared with before (see Table 3).

**TABLE 3:** Mean scores for learning before and immediately after the session (second questionnaire) with relevant qualitative themes: **Joint display**

Item	Pretest mean score (95% CI)	Post‐test mean score (95% CI)	*p*‐Value	Cohen's d	Theme
I understand the meaning of social determinants of health.	3.25 (2.73–3.77)	4.3 (3.99–4.61)	<0.001	0.83	
I understand how patients' social conditions may affect their health status.	3.85 (3.39–4.31)	4.3 (4.03–4.57)	0.035	0.40	
I feel positive about communicating with patients about their social conditions.	4.2 (3.75–4.65)	4.5 (4.22–4.78)	0.082	0.27	
I can describe situations where I should take detailed information about patients' social conditions.	2.65 (2.24–3.06)	4.1 (3.80–4.40)	<0.001	1.37	Shifting from patient characteristics to the view of a medical professional
I feel confident in my ability to gather information about patients' social conditions.	2.85 (2.54–3.16)	3.65 (3.33–3.96)	<0.001	0.86	Putting value on patients' preference and awareness of potential to overlook issues by sole use of the mnemonic
I understand that I need to coordinate healthcare to fit patients' social conditions.	3.95 (3.53–4.37)	4.55 (4.31–4.79)	0.002	0.60	Shifting from direct problem‐solving to prolonged involvement

Abbreviation: CI, Confidence interval.

The written comments in the second questionnaire indicated several changes in participants' approaches, which explained quantitative data. (1) Before the session, participants based their idea about whether or not to ask patients about social conditions mainly on patients' characteristics, which included deviation from normal consultation behavior or treatment course, and physical appearance associated with social deprivation. After the session, residents showed new ideas: they seek patients' social information when wondering what kind of care they should provide. (2) Participants accepted the checklist well and considered ‘patients’ preference and value’ to be especially important. Some participants suggested, however, that using the checklist alone could mean that they overlooked some aspects of patient‐specific social issues. (3) Before the session, participants' idea of how to coordinate care to fit patients' social conditions was limited to direct problem‐solving. After the session, participants recognized that they might not be able to solve problems immediately and that it was important to stay involved with patients over a long period (see Table 3).

### 3.3 Learning through daily practice and behavior

The results of analysis about learning through daily practice were divided into three categories: (1) reflection as a medical professional; (2) implementing new practices based on learning; and (3) concerns about addressing patients' social conditions (see Files 3–5 in Appendix S1 for codes and illustrative quotations).

#### 3.3.1 Reflection as a medical professional

Participants learned that lack of understanding of patients might have negative consequences. They realized that they had been imposing medical correctness and getting frustrated with patients who could not adhere to it. They also recognized that they often missed opportunities to understand patients and attached a stigma to patients in difficulty by not listening to their stories.

Participants considered what a doctor was and should be in facing social contexts. They gave a positive meaning to their practice about SDoH and suggested that residents had some unique advantages in addressing patients' social issues.

They recognized that the ability to address patients' social conditions was an acquired skill. They constructed an organic knowledge structure with related domains and held a future vision of how they could grow.

#### 3.3.2 Implementing new practices based on learning

Participants identified the assessment tool as putting social factors into a medical context. The tool could help them organize patients' complex social conditions concisely, measure the extent of their understanding of patients, identify what they could do to address patients' difficulties. They used the tool in different ways depending on the situation. When they had time to spare, they collected information to complete the sheet. When time was limited, such as in the emergency room, they used a simplified set of questions in their own way.

Participants had come to know and accept patients as they were. They became aware that patients live unique and colorful lives, even though the details of their lives are often overlooked in the medical context. They began to communicate with patients to understand them as individuals. Through this type of communication, participants began to respect patients' personalities and build good relationships. However, some participants expressed a sense of helplessness and futility. They were focused on short‐term problem solving (e.g., selection of discharge destination, decision about which services to introduce) and felt that there was nothing they could do as an individual doctor to directly solve these patients' social problems.

Participants recognized that their awareness of patients' social difficulties led to a deeper understanding and delivery of contextualized care. Some participants reported that when they encountered patients who seemed difficult to deal with, they now inferred that these patients might have hidden social problems, and aggressively gathered additional information to identify underlying social conditions. They did practice medical care that considered patients' social backgrounds. Their understanding of the diversity of patients' social backgrounds and their reduced cognitive load from knowing about a specific assessment method helped in this process.

Participants reported that their learning had added new value to their daily practices. Before the session, some participants had tended to consider that their role was exclusively to respond appropriately to patients' diseases and biomedical problems, especially in the emergency room. However, they now recognized that they also had a role in addressing emergencies in patients' social situations. They adopted a strategy of isolating patients' difficulties from their personalities and seeing these difficulties as challenges to be solved together with the patient, rather than seeing patients as a complex and troublesome whole. This enabled them to establish a therapeutic alliance with patients.

Participants considered the assessment tool as a platform for interprofessional collaboration and its advancement. They reported that by using the tool to collect and organize patient information (for example, summarizing information in the medical record), they were able to communicate smoothly with other interprofessional team members. Participants reaffirmed the need for multidisciplinary collaboration to deliver conceptualized care to patients.

#### 3.3.3 Concerns about addressing patients' social conditions

Some participants expressed concerns about using specific tools for asking about and assessing patients' social conditions. First, they were concerned that their biomedical evaluation would be ambiguous if they were concentrating on social issues. Second, they were afraid that patients would complain. Third, they were worried that patients would feel a loss of dignity if asked about their private lives. Fourth, they were concerned that comprehensive evaluation would take excessive time and effort.

## 4 DISCUSSION

Residents responded well to learning about SDoH and the assessment tool (RQ1). They appreciated that the tool would enable them to gather information about patients' social contexts, and they would therefore be able to deliver more suitable care and respect patients' wishes and values. They also recognized that they could not always reach immediate solutions and that staying involved for a long time was important.

Residents reflected on their previous experience as medical professionals and accepted patients as they were, through learning about SDoH (RQ2). Using the assessment tool in their clinical settings with some modifications encouraged them to put patients' social backgrounds into the context of medical care. It also enriched their understanding and care provision, added new value to their practice, and facilitated interdisciplinary collaboration. Overall, they viewed the tool positively as facilitating communication but expressed some concerns about its use (RQ3). Mixed‐methods approach conveyed an insight about residents' learning and behavior that was unexpected before this research.

There were several possible effects of learning about the SDoH assessment tool. First, the tool served as a guide for what to do in clinical practice. Simply learning about the theory of SDoH may make identification of patients' social issues daunting, and trainees can learn about the effective use of tools through practice.^27^ In this study, participants did not use the tool in its original form and were creative in modifying it to suit their situation, for example as a question guide. This finding suggests that situated learning might occur in their clinical settings: the tool, the residents, the environment, and their context and culture interacted with each other.^28^ We should consider the nonlinearity of these interactions when measuring the outcomes of SDoH education.

Second, the tool helped residents organize patients' information to match their medical contexts. Previous research reported that residents learning about SDoH and practicing assessing clinical scenarios using a worksheet would be better able to identify patients' needs but had difficulty in organization and questioning.^8^ Showing residents some specific ways to organize patient information may enable them to achieve a more effective educational outcome.

Third, the tool might reduce residents' cognitive load. Residents admitted that it was often difficult to assess both biomedical and social aspects of patients' situations because they were overwhelmed by the volume of work that needed to be accomplished during a patient encounter. Knowing what to do next made it easier to approach patients' social conditions.^29^ Cognitive load is determined by the number of information elements that must be processed simultaneously within a particular time.^30^ Using guided tools may help to reduce the problem‐solving required in clinical practice and lead to better outcomes.^31^ This finding seems consistent with previous research about patients' social needs and burnout in medical professionals. Although seeing patients with unmet social needs is burdensome and a lack of resources is associated with more symptoms of burnout,^32^, ^33^ having the capacity to meet patients' social needs may mitigate the burden.^32^, ^34^, ^35^

A traditional concern with asking patients about social issues is that it may lead to listening without appropriate intervention.^36^ One study reported that approximately half of residents who fully elicited and explored patients' social conditions did not take any further action.^27^ In this study, residents who sought immediate solutions to immediate problems felt limited in their roles and helpless. However, those who sought to understand their patients based on long‐term relationships reported richer practices. These residents anticipated and identified hidden social issues among ‘difficult’ patients and shifted their view from ‘problematic patients’ to ‘problems to address with patients’. This led to the establishment of a therapeutic alliance and interprofessional collaboration. These findings imply that learning about SDoH in clinical practice includes much more than simply screening and problem‐solving.

Our findings suggest that medical education about SDoH and its assessment may trigger a transformation in residents' clinical approaches. However, this study had several limitations. First, it only had a few participants from a relatively homogeneous group, all working in hospitals in the same federation. This may weaken the external validity of this study. Second, we assessed the participants' behavior only 1 and a half months after the program because of time constraints. We may therefore have missed behavioral changes requiring a longer period.^37^ Third, we did not measure patients' outcomes. Finally, the first author instructed the participants and interviewed them. Although this relationship could allow for a deeper understanding of the participants, the participants might make comments that they thought desirable for the first author.

### 4.1 Implications for future research

To collect quantitative data, we developed the questionnaire that is specific to this educational session via a literature review and an interview with experts. These steps secured its content validity to some extent. However, the evaluation was limited by the design of this study, including small sample size. In addition, clinical importance of the pre‐post score difference is unclear, because our questionnaire did not define minimally important changes. The values of Cohen's d in 4 out of 6 questions about learning exceeded 0.5, which is commonly considered as moderate effect size,^22^ and interpreted as positive changes in assessing residents' skills and confidence addressing SDoH in a previous study.^20^ However, these values did not always mean clinically important outcomes. Further research should be needed to develop a universal questionnaire for assessing competency for addressing SDoH. We have revealed unexpected residents' learning outcomes via qualitative analysis, and these findings may help develop a validated questionnaire.

To better understand these complex learning effects, we plan to perform larger‐scale, hypothesis‐testing quantitative studies. Further research is also needed to determine whether the use of assessment tools leads to a reduction in the cognitive load of medical professionals who address SDoH.

## CONFLICT OF INTEREST

The authors have stated explicitly that there are no conflicts of interest in connection with this article.

## Supporting information


Appendix S1
