# Supplementary material for: Experience of residents learning about social determinants of health and an assessment tool: Mixed‐methods research
Source: J Gen Fam Med. 2022 May 15;23(5):319–26. doi: 10.1002/jgf2.559 (PMC9444009; doi:10.1002/jgf2.559)
Supplement: Supplementary file 1 — Appendix S1 [file JGF2-23-319-s001.docx]

Supplemental File 1**.** Social vital signs sheet

| Social factors to ask^†^ | What: current conditions | Why: causes of current conditions | How: actions to take |
| --- | --- | --- | --- |
| Human network and relationships |  |  |  |
| Employment and income |  |  |  |
| Activities that make life worth living |  |  |  |
| Literacy and learning environment |  |  |  |
| Total physiological needs, including adequate food, shelter and clothing |  |  |  |
| Healthcare systems |  |  |  |
| Patient preference/values |  | | |

†The mnemonic “HEALTH+P” is used widely in Japanese primary care.

Supplemental File 2. Interview guide

1. What did you learn from the workshop?

1.1 What new knowledge did you gain?

1.2 What new perspectives have you gained?

1.3 What did you gain from practice using the SVS sheet?

2. How do you apply what you learned in the workshop to your clinical practice?

2.1 How do you apply it on the ward?

2.2 How do you apply it in outpatient clinics?

2.3 How do you apply it in case conferences?

3. What has changed in the way you deal with patients after the workshop?

3.1 Has the content of your conversation with patients changed?

3.2 Have the emotions that arise when you deal with patients changed?

Supplemental File 3. Themes and codes in the category of “Reflection as a medical professional”

| Theme | Codes | Illustrative quotations |
| --- | --- | --- |
| Negative consequence of lack of understanding of patients | Imposition of medical correctness and frustration with patients | I often felt that I wanted to provide the best medical care to my patients, or that they would be better off if they changed their behavior, and I was sometimes annoyed by thinking that they didn't listen to me, or that they should do better. |
|  | Missed opportunities to understand patients by preventing patients from telling their stories | I had seen many patients in the emergency room who evoked negative emotions for me, and I reflected on the fact that they might have some issues with their SDoH. |
| Doctor as an intervener in social contexts | Social perspective overwhelmed by biomedical assessment | There are so many biomedical matters that need to be assessed during the initial consultation... If I address all these matters, I will have no time to consider SVS. |
|  | Reconstructing the view of medicine | I think I’ve been naturally listening to patients’ social backgrounds. This session taught me the significance of my action and how to do better. |
|  | Advantages of being a trainee | Trainees can play a role in interviewing patients about their social background as well as seniors or supervisors and this is different from other topics. Trainees can think about what is best for the patient, ask patients for their fundamental values, and then contribute to the patients and medical staff team. I think trainees have advantages in addressing patients’ social conditions |
| Positive and concrete future visions | Improved ability to ask about social approaches perceived as skill acquisition | I found it surprisingly difficult to ask questions about what the patient really cares about while choosing the right phrases and watching the way patients talk and behave. I think learning about SVS and practicing in a daily setting has improved my skill to do this. |
|  | Building an organic knowledge structure with related domains | I had learned about difficult patient encounters before, so I grasped SVS in that context. |

Supplemental File 4. Theme and codes in the category of “Implementing new practices based on learning”

| Theme | Codes (L: level of learning, B: level of behavior) | Illustrative quotations |
| --- | --- | --- |
| Putting patients’ social backgrounds into a medical context | Learning concrete methods to organise complex social conditions in a concise manner (L) | SVS has allowed me to ask clinically meaningful questions when asking about patients’ social backgrounds. |
|  | Monitoring how well I understand patients (L) | If I only look at patients’ diseases, I immediately think that the diseases have been cured and the patient should be discharged. However, I might be able to gather enough information to fill out the SVS sheet from every patient if asking the right questions. |
|  | Identifying leverage points (B) | By writing on the SVS sheet, I could see that a lot of problems were being consolidated into one or two. Seeing the underlying problem, I understood what to do next. |
|  | Incorporating exploration of social factors into daily practice (B) | I think a framework for asking patients about social conditions has been established. This would help prevent oversights, just like asking patients in pain according to the mnemonic OPQRST.^†^ This framework is very meaningful for me. |
|  | Facilitating/deepening dialogue with patients guided by the tool (B) | The SVS sheet provided me with many perspectives. For example, I interviewed a patient who was reluctant to go to the emergency room. I found out that she had no means of transportation, thought that calling an ambulance would disturb the neighbors, and was financially distressed. I realised she was a person who cared about others a lot, and I asked her about her personal relationships with others. |
| Knowing patients as they are | Awareness of the inherent richness of patients’ lives that is discarded in the medical context (L) | Does this patient really need the treatment I thought was best? Is it really necessary for a patient whose life background is completely different from mine? I started to wonder about these questions. In doing so, the impatience I had felt before, when I wondered why some patients didn’t follow my instructions, subsided a little. |
|  | Rigid perspectives due to preoccupation with short-sighted problem-solving (L) | It is still quite difficult to coordinate every detail for the goal, considering where to discharge the patient, who will support them, and so on. |
|  | Communication to understand patients (B) | I thought it was important to know who they are as individuals, rather than as patients. I had never asked patients about their favorite activities or their values, but now I do. |
|  | Respect for the patient’s personality that emerges from a deeper understanding (B) | Learning more about patients has made me feel like they are more important to me. |
| Enriching understanding and care induced by awareness | Conviction and empathy gained by knowing about patients’ social conditions (L) | Before the session, I was upset because I had no idea why some patients neglected their diseases. Now, I know that they are actually doing everything they can to get out of the difficult situation they are in. For example, they may be working desperately hard to get out of poverty. |
|  | Estimating and identifying the presence of underlying social problems in patients who seem difficult to deal with (B) | Whenever I am annoyed by a patient, I now think about why this patient has come to the hospital, and their underlying needs. |
|  | Practicing patient-specific effective care (B) | I saw a woman in her 90s in the ER. Her medical condition was relatively stable, but the family wanted her to be hospitalised. Remembering the session, I listened to their stories. I found out that their lives were really difficult. In the end, I admitted the patient to the hospital. I am glad that I was able to make that decision. |
| Adding new value to daily practices | Finding a new role in addressing emergencies in social conditions (L) | I had thought that the emergency room was a place to efficiently pick up patients who were in danger because of their biomedical condition, but I discovered that it can also be a place to support patients with social difficulties. |
|  | Support for patients’ recovery by isolating their difficulties from their personalities (B) | Whenever I have antipathy or negative feelings towards a patient, I wonder how I could improve their condition and make myself less uncomfortable, and I began to think that if I could solve the patient’s problems, I would be less uncomfortable, and then the patient would be on the right track. |
|  | Establishing a therapeutic alliance based on good relationships (B) | I used to desperately try to intervene in all the problems of my patients, but when I reconsidered, I realised that there are things that we can do and things that we can’t. That’s why I decided to intervene where I can, and came to think that it is important to maintain an appropriate distance from patients. |
| Platform for interprofessional collaboration and its advancement | Gateway to interprofessional collaboration (B) | I knew that I couldn’t do it alone. All the information from various professions, such as social workers and nurses, is necessary. I think it is very important to use the SVS sheet to verbalise and share the problem to get interprofessional collaboration. |
|  | Lingua franca as a basis for interprofessional collaboration (B) | By using SVS, I was able to inform the ward nurses about the patient’s condition. This led to further progress in the conferences. Until then, I had not been able to share information well. |

†OPQRST: onset, provocation or palliation, quality, region and radiation, severity, time

Supplemental File 5. Themes in the category of “Concerns about addressing patients’ social conditions”

| Codes | Illustrative quotations |
| --- | --- |
| Concerns that biomedical evaluation would be ambiguous | I am concerned that the diagnosis and treatment of diseases may be neglected because of a preoccupation with patients’ social backgrounds. |
| Concerns that patients would complain | I feel like I can’t step in. I worry that I will be interfering too much with patients. |
| Concerns that patients will suffer loss of dignity | There are times when I feel that I need to ask patients about their social backgrounds, but I find it difficult to ask. This is because I feel that if I choose the wrong words or questions, I might upset them. |
| Concerns that the cost of evaluation would be excessive | I worry that it would be difficult to get the vast amount of content needed to complete the SVS sheet. |
